# Classification and Determination of Severity of Corneal Ulcer with Vision Transformer Based on the Analysis of Public Image Dataset of Fluorescein-Stained Corneas

**DOI:** 10.3390/diagnostics14080786

**Published:** 2024-04-09

**Authors:** Talha Burak Alakuş, Muhammet Baykara

**Affiliations:** 1 Faculty of Engineering, Department of Software Engineering, Kırklareli University, 39100 Kırklareli, Türkiye; 2 Faculty of Technology, Department of Software Engineering, Fırat University, 23119 Elazığ, Türkiye

**Keywords:** corneal ulcer, artificial intelligence, classification, vision transformer, deep learning

## Abstract

A corneal ulcer is a condition in which an injury to the corneal surface occurs as a result of infection. This can lead to severe vision loss and even blindness. For this reason, early diagnosis of this disease is of great importance. Deep learning algorithms are used in many critical health applications and are used effectively in the early diagnosis stages of diseases. Thus, a deep learning algorithm was applied in this study and corneal ulcer and severity were predicted. The study consisted of four stages over three different scenarios. In the first scenario, the types of corneal ulcers were predicted. In the second scenario, the grades of corneal ulcer types were classified. In the last scenario, the severity of corneal ulcers was classified. For each scenario, data were obtained in the first stage and separated according to the relevant labels. In the second stage, various image processing algorithms were employed, and images were analyzed. At this stage, the images were also augmented by various processes. In the third stage, ViT architecture, a new deep learning model, was used, and the images were classified. In the last stage, the performance of the classifier was determined by accuracy, precision, recall, F1-score, and AUC score. At the end of the study, the ViT deep learning model performed an effective classification, and accuracy scores of 95.77% for the first scenario, 96.43% for the second scenario, and 97.27% for the third scenario were calculated.

## 1. Introduction

Corneal ulcer is a disease that occurs as a result of inflammation of the cornea (the transparent layer covering the front surface of the eye) and scarring in the tissue. Corneal ulcers are usually caused by infectious (bacterial, viral, fungal, or protozoal), but other factors, such as trauma, severe dryness, or contact lens wear, can also contribute [1]. Symptoms and signs of a corneal ulcer include eye inflammation, painful eye, excessive tearing, blurred vision, white spot on the cornea, swollen eyelids, haze or eye discharge, sensitivity to light, and feeling like something is in the eye [2].

In order to diagnose corneal ulcer, an eye examination is performed. A slit-lamp microscope is used for the eye examination [3]. Measuring the severity of a corneal ulcer, particularly flaky ulcers, requires precise segmentation. While manual segmentation is seen as the most accurate method, it is also very time-intensive [4]. Several factors complicate the accurate segmentation of corneal ulcers, including notable differences in the appearance of point-flaky and flaky corneal ulcers, unclear boundaries, interference from noise, and a lack of dependable reference images from slit-lamp examinations. Different segmentation techniques are required to effectively identify and measure corneal ulcers in eye-staining images. The challenge is exacerbated by the diverse sizes and shapes of point-flaky mixed corneal ulcers and flaky corneal ulcers, making their segmentation in slit-lamp images particularly difficult [4]. In addition, the lack of high-quality datasets for corneal ulcers that would enable the use of segmentation algorithms, especially based on supervised learning, has hindered the development of such systems [5,6]. The segmentation process can be expressed as the first step used to detect and evaluate ocular surface damage. However, a computer-assisted self-diagnosis system can help specialists to localize and extract features from the corneal ulcer site for further evaluation. In this study, a deep-learning-supported application was developed to guide computer-aided systems, and the diagnosis and severity of corneal ulcer were determined.

Three different scenarios were considered within the scope of the study. In the first scenario, the types of corneal ulcers were predicted. In the second scenario, the grade of corneal ulcer types was analyzed, while in the third scenario, the severity of the corneal ulcers was determined. Four stages were designed for each scenario. In the first stage, the data were obtained, and the data were made ready by separating them into labels. In the second stage, various image processing algorithms were used. Accordingly, the blue channels were first removed from the images. Each of the images was then converted to gray color. Then, a Gaussian filter was applied, and images were blurred. Next, the Otsu thresholding method was employed, and the noises were removed from the images. Finally, masking was performed, and the information-containing feature was obtained from the images. After the analysis process, data augmentation was performed on the images. In this direction, four different methods were applied on each image: rotation, scaling, padding, and flipping. In the third stage, classification was performed with the ViT (Vision Transformers) deep learning model, which is a new deep learning model. In the last stage, the performance of the classifier was determined by the criteria of accuracy, precision, recall, F1-score, and AUC (area under the curve) score. The highlights of the study can be summarized as follows:

- For the first time in this study, corneal ulcers were classified and predicted by the ViT deep learning model.
- There are few studies in this field in the literature. With this study, a contribution has been made to the literature in this field, and the success of deep learning algorithms in detecting this disease has been demonstrated.
- In this study, in addition to other studies in the literature, the types and severity of corneal ulcers were also determined.
- With this study, it has been shown that artificial-intelligence-assisted diagnosis applications can help experts.

The rest of the study is organized as follows: In the Section 2, other studies in this field are examined and explained. In the Section 3, information is provided about the dataset, the applied image processing techniques, the methods applied for data augmentation, and the deep learning algorithm. Furthermore, the performance evaluation criteria of the deep learning algorithm are also included. In the Section 4, the results of the applications are explained, and the findings obtained from the applications for each scenario are displayed. Moreover, a discussion is presented, and the advantages and disadvantages of the study are mentioned. In the Section 5, the study is concluded and the contributions of the study to the literature are outlined.

## 2. Related Works

In this section, studies on corneal ulcers are reviewed and their results are presented. Although there are not many studies in the literature, it was observed that deep learning algorithms were used in various studies and corneal ulcers were predicted. In [4], the researchers developed a method for automatic detection of corneal ulcers. In the developed method, both image processing and deep learning methods were combined, and corneal ulcers were detected. In the study, image processing methods were evaluated to determine the borders and features of the corneal ulcer, while the deep learning model was used to detect the presence of corneal ulcers by using the learned features. The ResNet model was employed as a deep learning model and the performance of the classifier was measured with accuracy, precision, and recall values. At the end of the study, an accuracy score of 98.8% was obtained. In [7], the researchers predicted corneal ulcers using the modified VGG16 deep learning model. Image processing techniques were also used in the study, and masking, AHE (adaptive histogram equalization), rescaling, and normalization were used accordingly. After the data were prepared, they were classified and predicted with the modified VGG16, VGG16, and AlexNet deep learning algorithms. The performance of the classifiers was measured with accuracy, precision, recall, and F1-score values. At the end of the study, the highest accuracy score was obtained with the modified VGG16, and the result was calculated as 88.89%. In [8], the CNN (convolutional neural network) deep learning model was used, and the regions of corneal ulcers were determined. First, a heat map was applied to the images, and the distinction between important and unimportant regions was made. Then, the VGG16 deep learning model was applied, the images were classified, and the corneal ulcer regions were predicted. The performance of the classifier was determined by accuracy, kappa, and AUC values. At the end of the study, an accuracy score of 92.73% was observed. In [9], corneal diseases were detected from the eye images obtained by a slit-lamp microscope, and a deep learning algorithm was used. ResNet34, DenseNet, Inception-V3, and Ensemble were used as deep learning models. The performances of the classifiers were determined by accuracy, precision, recall, and AUC scores. At the end of the study, an AUC score of 0.89 was obtained with Ensemble.

Although there are few studies in this field in the literature, the achievements obtained from deep learning algorithms have shown that these algorithms can be applied effectively in this field.

## 3. Material and Methods

In this section of the study, information about the dataset and methods is provided. The dataset, the number of data points, and technical information about the data are presented. In addition, information about the ViT architecture applied in this study is provided, as well as information about the applied image processing techniques. Finally, the evaluation criteria of the classifier are also discussed.

### 3.1. Cornea Ulcer Dataset

The SUSTech-SYSU dataset was used in the study [10]. There are 712 fluorescein staining images in total in the dataset. Images were obtained from the Zhongshan Center for Ophthalmology at Sun Yat-sen University. There is no information concerning factors such as age, gender, and ulcer cause in the dataset. Within the scope of the study, images were analyzed over three different scenarios. In the first scenario, the types of corneal ulcers were predicted, while in the second scenario, the grades of corneal ulcer types were determined. In the last scenario, the severity of corneal ulcers was specified.

**Figure 1:**
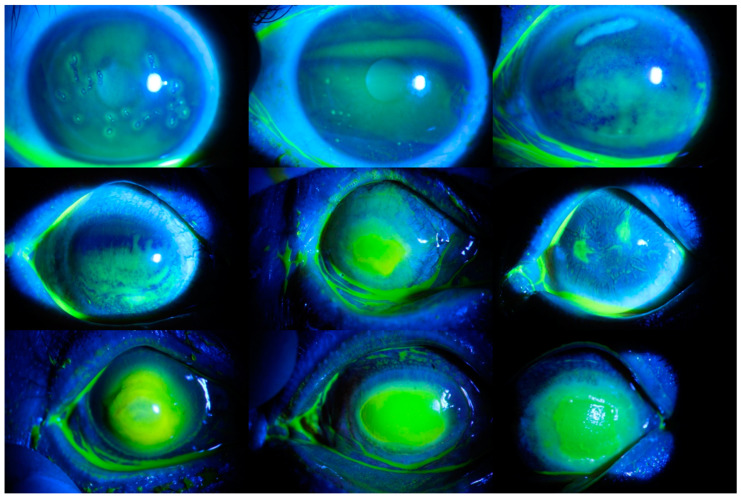
Various images of each type of corneal ulcer. The images in the first row represent point-like corneal ulcers, those in the second row represent point-flaky mixed corneal ulcers, and those in the third row represent flaky corneal ulcers.

Corneal ulcers were examined in three categories according to their shape and distribution characteristics: point-like corneal ulcers, point-flaky mixed corneal ulcers, and flaky corneal ulcers. A point-like corneal ulcer is the most common and is a relatively milder type. It is usually seen in the early stage of an eye infection. In such corneal ulcers, multiple small ulcer points are usually seen, and a definite pattern is observed [11]. The most dangerous corneal ulcer is the flaky corneal ulcer. In this type, the ulcer area is usually green in color and has a clear border. Sores form on the surface of the eye, and the patient’s vision is significantly reduced. In addition, it may cause vision loss [12]. Point-flaky mixed corneal ulcer is a corneal ulcer disease whose severity is usually between the other two types mentioned. In this type, an irregular distribution is seen, and both point-like and flaky ulcers are observed. In the first scenario, these types were used, and classification was carried out. There were 358 point-like corneal ulcers, 263 point-flaky mixed corneal ulcers, and 91 flaky corneal ulcers in the dataset. Various images of each category are presented in Figure 1.

**Figure 2:**
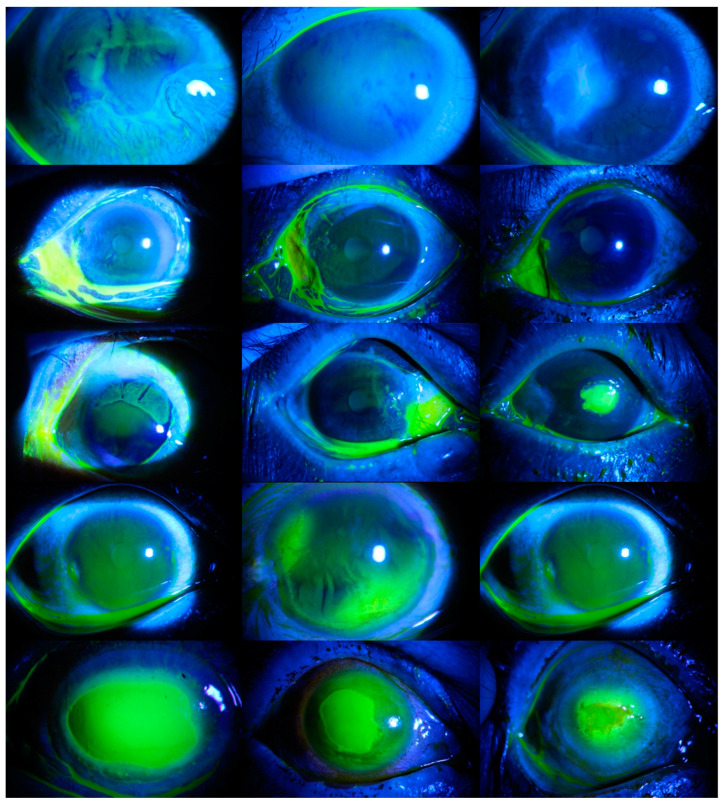
Various images of each type of corneal ulcer. Images belonging to without ulcer category are presented in the first row, images belonging to the micro-punctate ulcers category are in the second row, images belonging to the macro-punctate ulcers category are in the third row, images belonging to the coalescent macro-punctate ulcers category are in the fourth row, and images belonging to the ulcers with a patch value greater than 1 mm category are in the fifth row.

Within the scope of the second scenario, classification was performed according to the TG (Type Grade) rating. TG rating is a common staining grading method used worldwide. It consists of two different components. In the first component, the pattern is determined by the type of cornea, and in the second component, the severity of the corneal ulcer is observed [13]. Within the scope of this scenario, five different categories were determined. Images without ulcers were evaluated in the first category, micro-punctate ulcers in the second category, macro-punctate ulcers in the third category, coalescent macro-punctate ulcers in the fourth category, and ulcers with a patch value greater than 1 mm in the fifth category. There were 36 images in the first category, 78 images in the second category, 40 images in the third category, 10 images in the fourth category, and 548 images in the fifth category. In Figure 2, images of corneal ulcers belonging to each category are provided.

**Figure 3:**
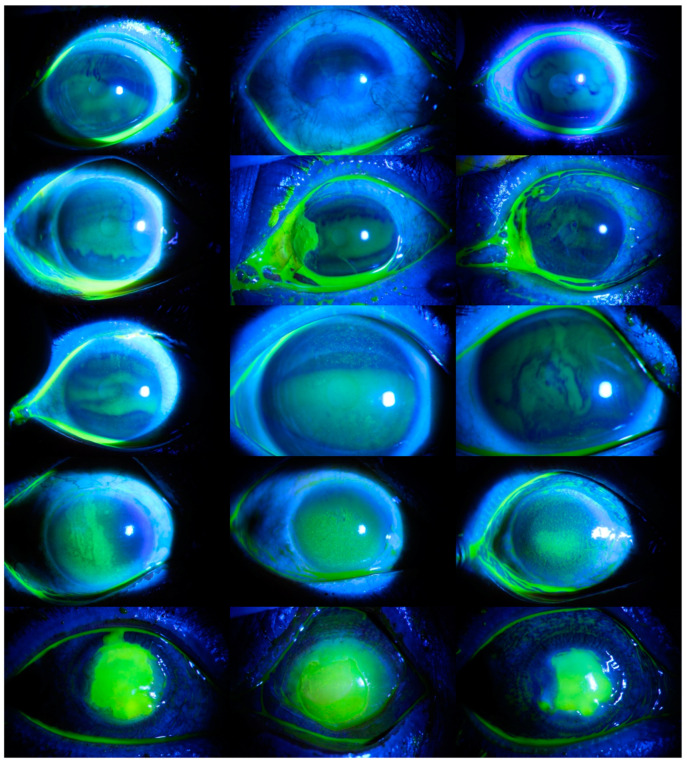
Various images of each type of corneal ulcer. Images belonging to the without ulcers category are presented in the first row, images belonging to ulcers surrounding no more than 25% of the cornea category are in the second row, images belonging to the ulcers surrounding up to 50% of the cornea category are in the third row, images belonging to the ulcers surrounding at least 75% of the cornea category are in the fourth row, and images belonging to the ulcers surrounding the center of the cornea category are in the fifth row.

In the third scenario, the severity grade was evaluated, and the severity of the corneal ulcers was classified. As in the second scenario, there are five categories in this scenario. Images without ulcers were evaluated in the first category, ulcers surrounding no more than 25% of the cornea in the second category, ulcers surrounding up to 50% of the cornea in the third category, ulcers surrounding at least 75% of the cornea in the fourth category, and ulcers surrounding the center of the cornea in the fifth category. There were 36 images in the first category, 98 images in the second category, 203 images in the third category, 273 images in the fourth category, and 102 images in the fifth category. In Figure 3, images of corneal ulcers belonging to each category are presented.

### 3.2. Image Processing Techniques

**Figure 4:**
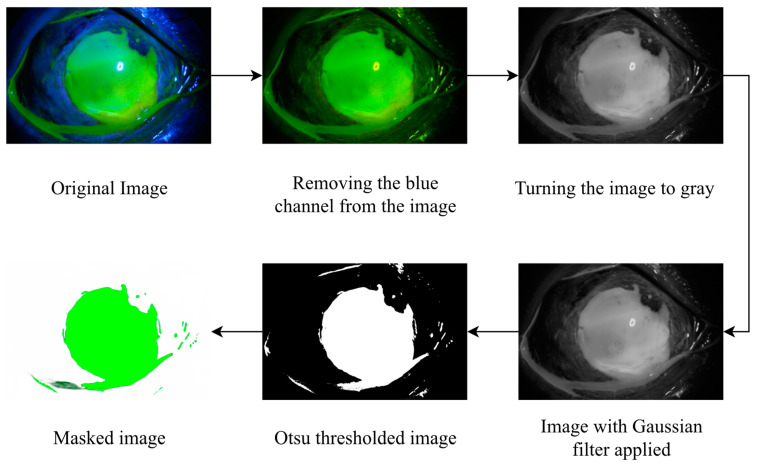
Application of image processing methods to a sample image.

The images in this study were not used as raw images—they were subjected to various preprocessing steps. In this direction, first, the blue channels on the images were extracted. Then, the images were converted to a gray color and the brightness values were determined. Next, the images were blurred by applying a Gaussian filter, and unnecessary information of the images was transferred to the background. Then, Otsu thresholding was performed, and the noise in the images was removed. Finally, the diseased areas were masked and made clear by applying a green color. These operations, performed on a sample image, are shown in Figure 4.

All the image processing techniques mentioned were applied to each image, and information-containing features were obtained from the images.

**Figure 5:**
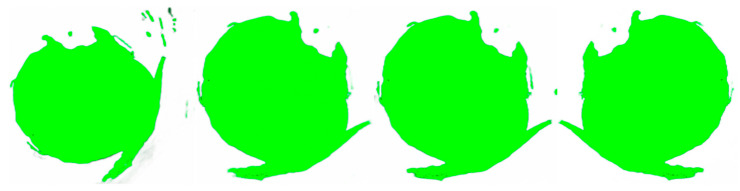
Augmented masked images. Respectively showing the image rotated 45 degrees, the padded image, the scaled image, and the flipped image.

The number of images is important in deep learning applications [14]. There were 712 images in total in the dataset, and this was not enough for the deep learning algorithm to be effective. Therefore, data augmentation was performed on the masked images, and rotation, scaling, padding, and flipping processes were employed. The masked and augmented images are shown in Figure 5.

After the data augmentation process, the number of images increased to 3560 (712 × 5), and they were made ready for classification.

### 3.3. ViT Deep Learning Model

**Figure 6:**
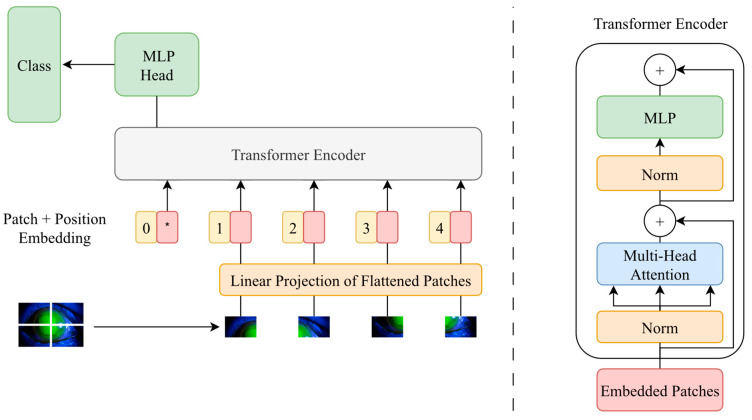
Working principle of the ViT deep learning model.

Deep learning is a sub-branch of machine learning and is a method based on ANNs (artificial neural networks). It stands out for its ability to achieve impressive results, especially when working on large datasets. Deep learning algorithms have been successful in many fields, especially in classification [15,16], image processing [17,18], natural language processing [19,20], voice recognition [21,22], and bioinformatics [23,24]. However, the use of deep learning methods requires large amounts of training data and computational power, and problems arise with model interpretability. Therefore, various types and more optimized deep learning models are currently being developed. The ViT deep learning model is one of the most recently developed models. It exhibits a high performance in image classification studies. The ViT model classifies images using the transformer architecture, which is successful in the field of NLP (natural language processing) [25]. In the ViT deep learning model, images are divided into patches of fixed size, and the pixel values of each patch are made into a flat vector. These vectors are then used as input to the transformer architecture. The transformer architecture enables the model to show better classification performance by learning the relationships between different patches in the image, thanks to its attention mechanism [26]. The schematic of the ViT deep learning model is presented in Figure 6.

There are several situations where the ViT deep learning model has advantages over traditional CNN models. These can be summarized as follows:

- Although the ViT deep learning model is effective on large datasets, it can be adapted to smaller and specific areas. In this way, the transfer learning capability of the model improves, and faster and better results can be obtained for new tasks [27].
- The transformer architecture is simpler, as opposed to traditional CNN architectures. This allows for easier understanding and adaptation of the ViT deep learning model [28].
- The transformer architecture can scale better with large datasets and images. In this way, it is effective for applications that work with higher-resolution images [27].

As a result, the ViT architecture is expressed as a significant advancement in the fields of deep learning and computer vision. This model is recognized as a powerful alternative to traditional CNNs, offering better classification performance and wide application areas [28]. Therefore, in this study, the newly developed ViT deep learning model, which has not been applied to the field before, was employed instead of traditional CNN architectures.

### 3.4. Evaluation Criteria

In this study, the performance of the ViT deep learning model was determined by the evaluation criteria of accuracy, precision, recall, F1-score, and AUC. All these values can be obtained from the confusion matrix. The confusion matrix is a kind of table used to show the performance of a classification algorithm. There are four different parameters in the confusion matrix: TP (true positive), TN (true negative), FP (false positive), and FN (false negative). The TP expression indicates that the predicted value is positive and correct, the TN expression indicates that the predicted value is negative and correct. In addition, FP indicates that the value is positive but false, while FN indicates that the value is negative and false. Accuracy, F1-score, precision, and recall values are based on the calculation of these given expressions with various equations. The calculations of these operations are shown in Equations (1) to (4):

(1) $$Accuracy=\frac{TP+TN}{TP+TN+FP+FN}$$

(2) $$Precision=\frac{TP}{TP+FP}$$

(3) $$Recall=\frac{TP}{TP+FN}$$

(4) $$F1\text{-}score=2*\left(\frac{Precision*Recall}{Precision+Recall}\right)$$

In addition, the AUC score is also an important evaluation criterion. The AUC score usually indicates how good the model is at separating classes. It is also used to determine the best threshold [29]. Interpretation of the AUC score is shown in Table 1.

**Figure 7:**
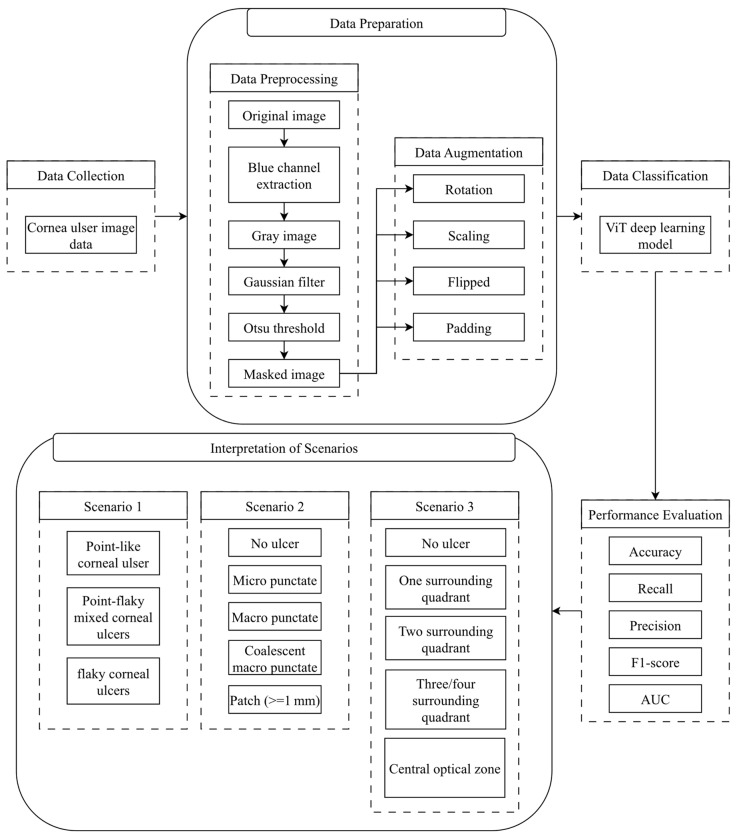
Flow chart of the study.

An AUC score of 0.49 and below indicates that the classification process was not performed. In addition, if the AUC score is between 0.50 and 0.69, it is considered a poor classification. An AUC score between 0.70 and 0.79 indicates an acceptable classifier, while a value between 0.80 and 0.89 indicates great classification. Values between 0.9 and 1 indicate that an outstanding classification has been obtained. The flow chart of the study is provided in Figure 7.

## 4. Application Results and Discussion

In this study, corneal ulcer prediction was carried out employing the ViT deep learning model. The performance of the deep learning model was determined by accuracy, precision, recall, F1-score, and AUC score. In the validation stage of the model, 10-fold cross-validation was applied. The study was performed over three different scenarios. In the first scenario, the types of corneal ulcers were predicted. In the second scenario, the grades of corneal ulcer types were determined and classified. In the third scenario, the severity of corneal ulcers was determined.

### 4.1. Classification of Corneal Ulcer Types

Here, we outline the first scenario realized within the scope of this study. Types of corneal ulcers were classified in this scenario. There are three different classes of corneal ulcers. These are: point-like corneal ulcer, point-flaky mixed corneal ulcer, and flaky corneal ulcer. For this reason, a three-class classification process was carried out. The classification results for this scenario are presented in Table 2.

**Figure 8:**
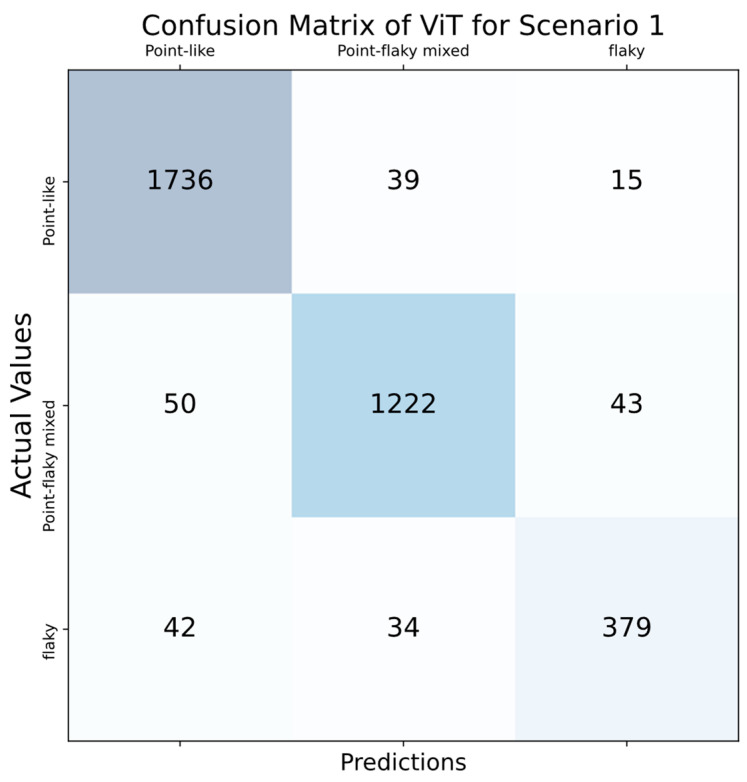
Confusion matrix for scenario 1.

Looking at the results in Table 2, the accuracy score was over 90% in all folds and the highest accuracy score was obtained in the second fold, with 98.05%. Moreover, almost all AUC scores were above 0.9. The highest AUC scores were obtained in both the 6th and 8th folds, and the result was 0.96. Considering the average values, an average of 95.77% accuracy and an average AUC score of 0.92 were obtained with the ViT deep learning model. A confusion matrix is used to assess the performance of a classification model. This matrix includes both the actual class labels and the predicted class labels generated by the model. It plays a crucial role in calculating various performance metrics for a classification model, such as sensitivity, specificity, and accuracy. By revealing the true positives, true negatives, false positives, and false negatives, the confusion matrix provides a detailed analysis of the model’s performance, showcasing how accurately it predicts different classes. This analysis is essential for understanding the strengths and weaknesses of the classification model in addressing specific class predictions. The confusion matrix was used in this study, and the performance of the model in each classifier was observed. The confusion matrix calculated for scenario 1 is shown in Figure 8.

Based on the confusion matrix shown in Figure 8, the developed ViT model accurately identified 1736 out of 1790 images in the point-like ulcer category, achieving an accuracy rate of 96.98%. Furthermore, it classified 39 images as point-flaky mixed, while the remaining 15 images were categorized solely as flaky. For the point-flaky mixed class, the classifier accurately assessed 1222 out of 1315 images, achieving an accuracy rate of 92.92%. Within this category, 50 images were identified as point-like ulcers, and the remaining 43 images were recognized as flaky ulcers. In the last category, the flaky ulcer class, 379 out of 455 images were accurately identified, achieving an accuracy rate of 83.30%. In addition, 42 images were classified as point-like, while the remaining 34 images were evaluated as point-flaky mixed ulcers. The outcomes demonstrate that the suggested deep learning model achieved an accuracy exceeding 90% in both the point-like and point-flaky mixed classes, whereas its performance was under 85% accuracy in the flaky class. Even though the accuracy rate did not hit the 90% mark in the last category, the overall results suggest that the classifier’s performance can still be considered successful. Balanced accuracy improves upon the traditional accuracy metric by adjusting for performance in imbalanced datasets, addressing one of the significant drawbacks of using the standard accuracy measurement. This makes it a more suitable alternative to conventional accuracy in many cases. For a classifier to be considered effective, its balanced accuracy score must be at least 0.9. In the given scenario, the classifier achieved a balanced accuracy of 93.75%, marking it as an efficient classifier.

**Figure 9:**
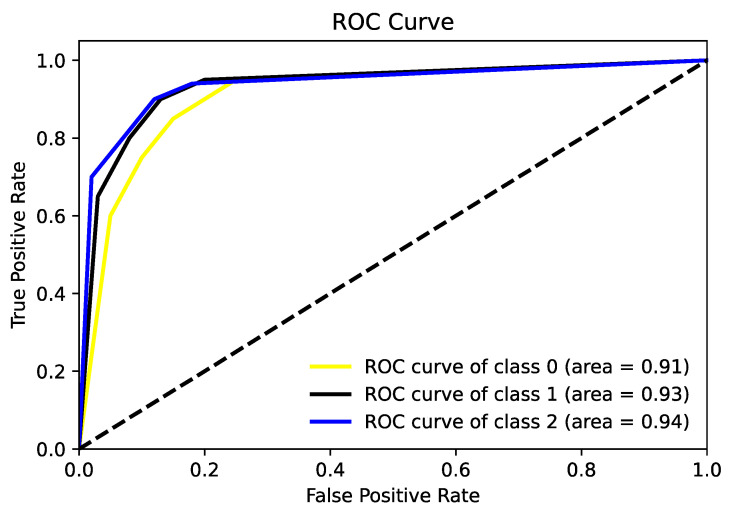
ROC plot calculated for the first scenario.

The ROC (receiver operating characteristics) curve serves as a statistical method, widely applied in areas such as medical diagnostics and machine learning to assess the efficacy of classification models across various threshold settings. It illustrates the connection between a model’s true positive rate (sensitivity) and its false positive rate (1—specificity), enabling an evaluation of performance differences at multiple thresholds. In this study, ROC curves were used, and the performance of the classifier at different thresholds was shown for each scenario. Figure 9 shows the average ROC plot of the classification process for the first scenario.

### 4.2. Classification of Grades of Corneal Ulcer Types

In the second scenario performed within the scope of this study, grades of corneal ulcer types were classified. Five different grades of corneal ulcer type were evaluated: absence of corneal ulcer, micro-punctate corneal ulcer, macro-punctate corneal ulcer, coalescent macro-punctate corneal ulcer, and ulcers with a patch value greater than 1 mm. For this reason, a five-class classification process was carried out. The classification results for this scenario are presented in Table 3.

**Figure 10:**
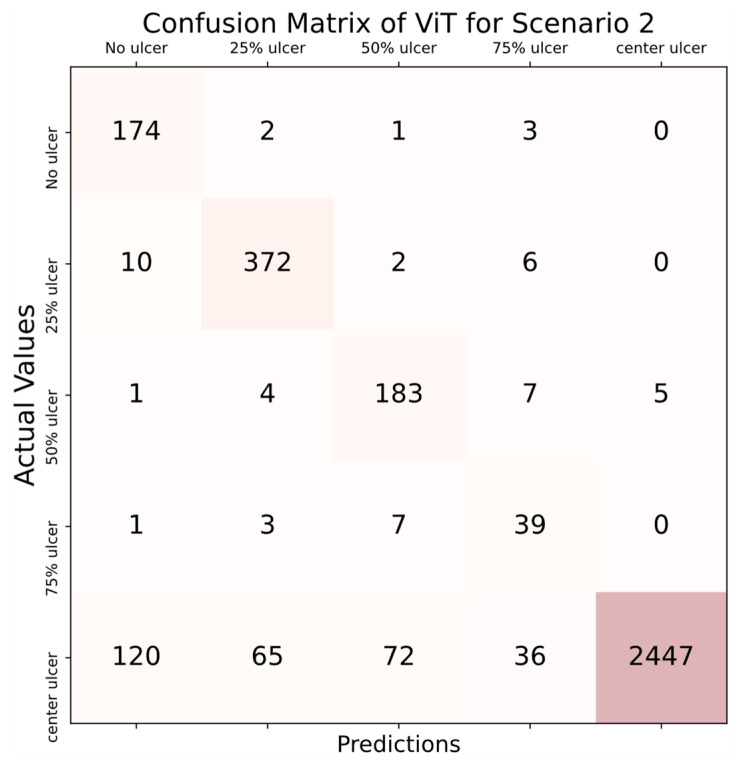
Confusion matrix for scenario 2.

When the results in Table 3 were examined, the accuracy score in all folds was over 90%, as in scenario 1, and the highest accuracy score was obtained in the 10th fold, with 97.87%. In addition, all AUC scores also performed above 0.9. The highest AUC scores were obtained in the 1st, 4th, and 6th folds, and the result was 0.94. Considering the average values, an average of 96.43% accuracy and an average AUC score of 0.93 were obtained with the ViT deep learning model. The confusion matrix computed for scenario 2 is presented in Figure 10.

According to the confusion matrix depicted in Figure 10, the implemented model correctly recognized 174 out of 180 images in the “no ulcer” category, resulting in an accuracy rate of 96.66%. Additionally, the model assigned 2 images to the 25% ulcer category, 1 image to the 50% ulcer category, and the remaining 3 images were exclusively categorized as 75% ulcer. In the 25% ulcer category, the classifier accurately evaluated 372 out of 390 images, resulting in an accuracy rate of 95.38%. Within this category, 10 images were misclassified as having no ulcers, 2 images were assigned to the 50% ulcer category, and the remaining 6 images were solely categorized as 75% ulcer. In the 50% ulcer category, the classifier accurately identified 183 out of 200 images, resulting in an accuracy score of 91.50%. Within this class, 1 image was mistakenly assessed as having no ulcers, 4 images were misclassified as 25% ulcer, 7 images were incorrectly categorized as 75% ulcer, and the remaining 5 images were erroneously labeled as completely ulcer (center). In the fourth category, the 75% ulcer class, the classifier accurately identified only 39 out of 50 images, resulting in a classification accuracy of 78%. Among the misclassified images, 1 was erroneously labeled as having no ulcers, 3 were incorrectly categorized as 25% ulcer, and 7 were inaccurately assessed as 50% ulcer. In the last category, the center ulcer class, 2447 out of 2740 images were accurately identified, achieving an accuracy rate of 89.31%. Within this category, 120 images were identified as having no ulcers, 65 images were classified as 25% ulcer, 72 images were labeled as 50% ulcer, and 36 images were recognized as 75% ulcer. The results indicate that the proposed deep learning model achieved an accuracy of nearly 90% in four distinct classes, while its performance dropped to below 80% accuracy in the fourth category. Despite not reaching the 90% accuracy threshold in the fourth category, the overall results indicated that the classifier’s performance can be deemed successful. In this specific scenario, the classifier attained a balanced accuracy of 93.92%, signifying its efficiency.

**Figure 11:**
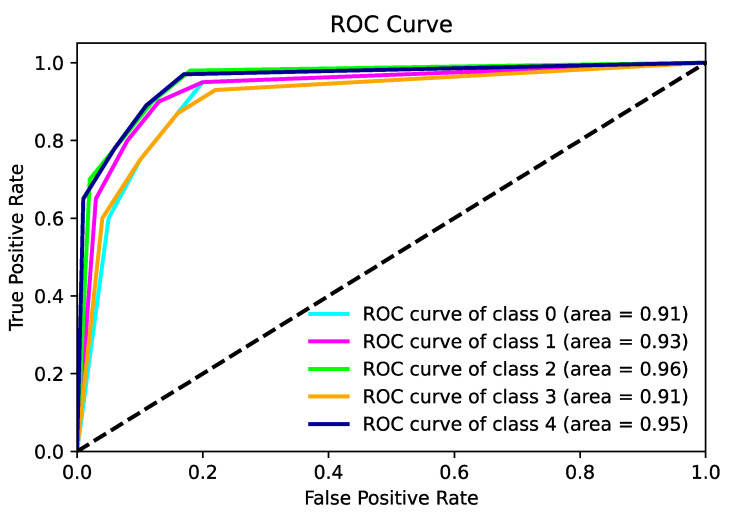
ROC plot calculated for the second scenario.

Figure 11 shows the average ROC plot of the classification process for the second scenario.

### 4.3. Classification of Corneal Ulcer Severity Grades

In the third and last scenario carried out within the scope of this study, the grade of corneal ulcer severity was classified. There are five different corneal ulcer severities. These were expressed as: absence of corneal ulcer, presence of corneal ulcer surrounding 25% of the cornea, presence of corneal ulcer surrounding 50% of the cornea, presence of corneal ulcer surrounding at least 75% of the cornea, and corneal ulcers surrounding the center of the cornea. For this reason, a five-class classification process was performed. The classification results for this scenario are presented in Table 4.

According to the results in Table 4, the accuracy score was over 95% in all folds, and the highest accuracy score was obtained in the 1st fold, with 98.93%. In addition, all AUC scores also performed above 0.93. The highest AUC score was obtained in the 1st fold, and the result was 0.96. Considering the average values, an average of 97.27% accuracy and an average AUC score of 0.95 were obtained with the ViT deep learning model.

**Figure 12:**
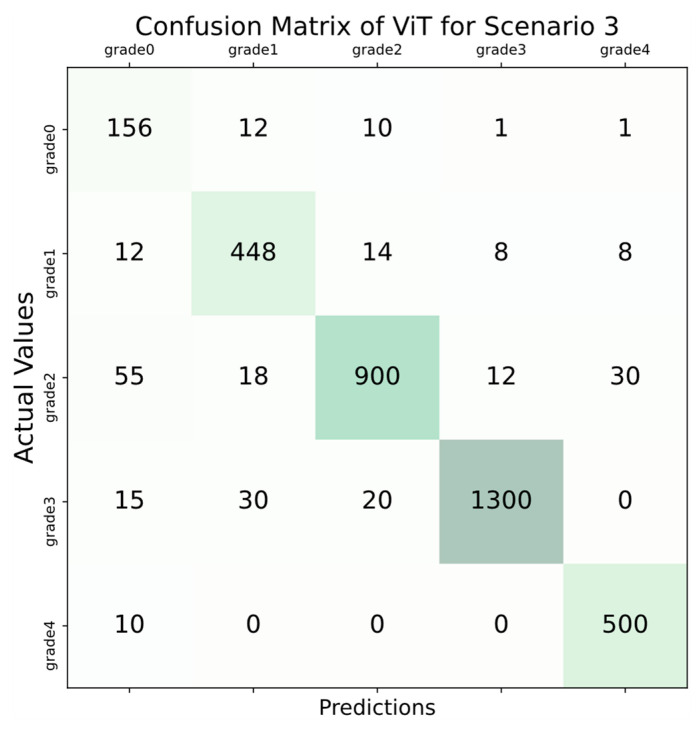
Confusion matrix for scenario 3.

The confusion matrix calculated for scenario 3 is displayed in Figure 12.

Based on the confusion matrix illustrated in Figure 12, the implemented model accurately identified 156 out of 180 images in the grade 0 category, achieving an accuracy rate of 86.66%. Furthermore, the model allocated 12 images to the grade 1 category, 10 images to the grade 2 category, and the remaining 2 images were exclusively classified as grade 3 and grade 4 categories. In the grade 1 category, the classifier correctly assessed 448 out of 490 images, achieving an accuracy rate of 91.43%. Within this category, 12 images were incorrectly classified as grade 0, 14 images were erroneously assigned to the grade 2 category, and the remaining 16 images were exclusively categorized as grade 3 and grade 4. In the grade 2 category, the classifier achieved an accuracy of 88.67% by correctly identifying 900 out of 1015 images. However, within this class, there were misclassifications, including 55 images wrongly assessed as grade 0, 18 images mislabeled as grade 1, 12 images incorrectly categorized as grade 3, and 30 images inaccurately labeled as grade 4. In the grade 3 category, the classifier achieved a classification accuracy of 95.24% by correctly identifying 1300 out of 1365 images. Nonetheless, among the misclassified images, 15 were erroneously labeled as grade 0, 30 were incorrectly categorized as grade 1, and 20 were inaccurately assessed as grade 2. In the grade 4 category, the classifier achieved an impressive accuracy rate of 98.04% by correctly identifying 500 out of 510 images. Only 10 images within this category were misclassified and incorrectly labeled as grade 0. Upon reviewing the results, it is evident that the accuracy scores for the grade 1, grade 3, and grade 4 classes surpassed 90%, whereas this rate stayed below 90% for the other classes. Despite certain classes not achieving accuracy above 90%, a holistic evaluation of the results suggested the classifier’s effectiveness. A compelling indication of this is the balanced accuracy score exceeding 90%, reaching 95.04%.

**Figure 13:**
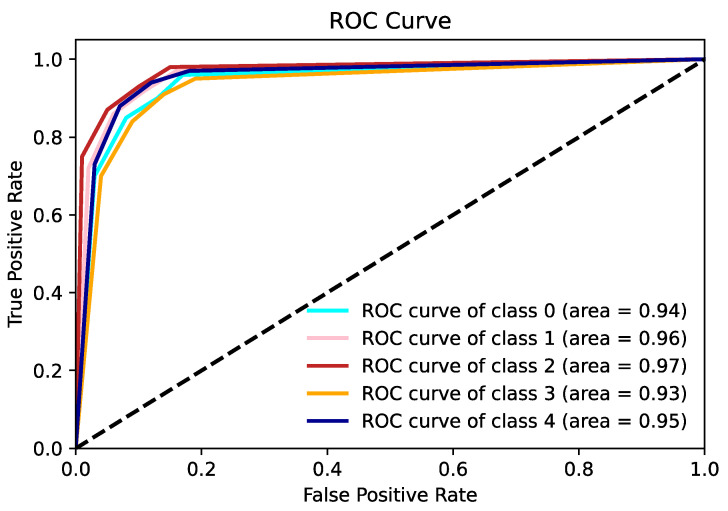
ROC plot calculated for the third scenario.

Figure 13 shows the ROC plot of the classification process for the third scenario.

### 4.4. Discussion

In this study, corneal ulcers were classified using images of eyes. Three different scenarios were evaluated and analyzed. When the first scenario was examined, an average of 95.77% accuracy and an average AUC score of 0.92 were obtained with the ViT deep learning method. When examined according to the AUC score, it was observed that the classifier achieved an outstanding classification. In scenarios 2 and 3, unlike the 1st scenario, the number of classes increased, and a five-class structure was used in both the 2nd and 3rd scenarios. In scenario 2, an average of 96.43% accuracy was achieved, while the average AUC score was 0.93. In scenario 3, these performances increased even more, and an average of 97.27% accuracy was calculated, while an average AUC score of 0.95 was observed. In both scenarios, an outstanding classification was achieved because the AUC score was above 0.90. With the increase in the number of labels, an increase in the performance of deep learning algorithms can be observed [31]. The increase in the number of labels in scenarios 2 and 3 increased the classification performance. Furthermore, various image processing techniques were applied before the classification process, and the images were made ready for classification. It is known that image processing techniques have positive effects on classification with deep learning [32,33,34]. In this study, operations, such as thresholding, masking, and noise removal, on images increased the performance of the classifier. Besides, the amount of data was increased in the study, and various data augmentation processes were applied for each image. It is known that deep learning algorithms are more effective with data augmentation in image processing [35,36]. In this study, increasing the images positively affected the classification performance. The comparison results are provided in Table 5.

From the results in Table 5, it is observed that the data augmentation process was effective for all scenarios. In the classification process performed without data augmentation, accuracy scores of 87.56% for the 1st scenario, 88.23% for the 2nd scenario, and 88.65% for the 3rd scenario were observed. While the accuracy score did not exceed 90% in the scenarios performed without data augmentation, the accuracy scores increased above 90% after data augmentation. Similarly, in the classification process performed without data augmentation, the AUC score remained below 0.90 in all scenarios and performed great classification. However, after data augmentation, the AUC scores rose above 0.90 and performed outstanding classification. In summary, data augmentation had a positive effect on the performance of the classifier.

In this study, a new deep learning model, ViT, was used instead of traditional CNN architectures. Although there are not many studies in this area in the literature, current studies have performed traditional CNN-based classification. In Table 6, the results from studies in the literature and those obtained in this study are presented. Studies in the literature only carried out the first scenario.

In the studies in Table 6, data augmentation was performed, and various image processing techniques were used. Examining the results in Table 6, the proposed study was more effective than that in [7,8], but less effective than that in [4]. One of the main reasons for this may be the development of a new masking method and its adaptation to this field in the study in [4]. Note that the methods used are all traditional CNN-based methods. However, studies [7,8] could not catch up with the performance of the ViT deep learning algorithm. The fact that the ViT deep learning model is more scalable, has long-range dependencies, has a more flexible architecture, and is based on less feature engineering makes it more effective than traditional CNN architectures [27,28].

As in every study, there are various limitations and advantages in this study. These can be listed as follows:

- A new deep learning model, ViT, was used, and the performance of this model was evaluated in image analysis. This model, which was adapted for the first time in this field, showed an effective performance.
- Despite the small amount of data used, data augmentation was performed, and successful results were obtained.
- The performance of image processing methods over biomedical images was reinforced again, and the results were observed.
- While there were originally 712 images in the study, this was increased to 3560 images. Although the classification process with these images was successful, these are synthetic images. A classification process based on real images will lead to healthier results.
- Some image processing techniques were used in this study. There are many image processing techniques, and more effective results could be observed using different techniques.
- The ViT deep learning model was used for classification purposes in this study. It can be used for feature extraction, combined with various deep learning and machine learning models, and better results could be observed.
- The effectiveness of machine learning and deep learning models is heavily reliant on the quality and representativeness of the utilized datasets. This is because any shortcomings, errors, or biases present in the datasets can profoundly impact the precision, ability to generalize, and trustworthiness of these models’ outcomes. In health-based applications, assembling datasets and uncovering their attributes is notably difficult. Consequently, it is imperative for experts to generate and share datasets of higher quality for open access. Doing so will underscore their importance in assessing the performance of existing machine learning and deep learning models and facilitate the creation of more robust models.

## 5. Conclusions

In this study, corneal ulcers were predicted, and this procedure was performed over three different scenarios. The study consisted of four stages. In the first stage, data were obtained, and images of the corneal ulcer were collected. In the second stage, various image processing techniques were applied on the images, and the data were augmented to prepare them for classification. In the third stage, a new deep learning model, ViT, was used, and the images were classified. In the final stage, the performance of the classifier was determined by accuracy, precision, recall, F1-score, and AUC score. All stages were designed the same for all scenarios. In the first scenario, the types of corneal ulcer were predicted, and a three-class structure was used. As a result of the classification, an average of 95.77% accuracy was obtained, while an average AUC score of 0.92 was observed. In the second scenario, the grades of corneal ulcer types were classified, and a five-class structure was created. As a result of this classification, an average of 96.43% accuracy and an average AUC score of 0.93 were obtained. In the last scenario, the grades of corneal ulcer severity were classified, and a five-class structure was created, as in the second scenario. The highest classification process was obtained in the third scenario, with average accuracy and AUC scores of 97.27% and 0.95, respectively. In this study, the ViT deep learning model was used for the first time in the classification and prediction of corneal ulcers. Biomicroscope measurements, while being relatively quick and cost-effective, can yield images that are often challenging to interpret with certainty. In this context, machine learning and deep learning techniques can assist clinicians in making accurate diagnoses more effortlessly and reliably. Furthermore, critical diseases, such as corneal ulcers, are diseases for which early diagnosis is important. Through this study, it has been shown that artificial-intelligence-supported systems that can help experts can be developed.

## Figures and Tables

**Table 1:** Interpretation of the AUC score [30].

AUC Score	Explanation
0.00–0.49	No distinction
0.50–0.69	Poor classification
0.70–0.79	Acceptable classification
0.80–0.89	Great classification
0.90–1.00	Outstanding classification

**Table 2:** Classification results of scenario 1.

Fold	Accuracy	Precision	Recall	F1-Score	AUC
Fold 1	96.66%	95.78%	96.76%	96.27%	0.90
Fold 2	98.05%	97.60%	98.42%	98.00%	0.90
Fold 3	93.23%	93.44%	93.82%	93.63%	0.93
Fold 4	95.97%	94.76%	94.38%	94.57%	0.91
Fold 5	93.38%	94.28%	92.78%	93.52%	0.93
Fold 6	95.83%	96.75%	96.60%	96.67%	0.96
Fold 7	97.53%	96.01%	97.55%	96.77%	0.91
Fold 8	97.60%	95.95%	98.10%	97.01%	0.96
Fold 9	94.64%	94.13%	98.20%	96.12%	0.94
Fold 10	94.83%	97.34%	93.11%	95.18%	0.90
**Average**	**95.77%**	**95.60%**	**95.97%**	**95.78%**	**0.92**

**Table 3:** Classification results of scenario 2.

Fold	Accuracy	Precision	Recall	F1-Score	AUC
Fold 1	96.71%	97.83%	96.89%	97.36%	0.94
Fold 2	94.31%	93.51%	95.03%	94.26%	0.93
Fold 3	97.18%	96.48%	94.31%	95.38%	0.92
Fold 4	97.86%	96.56%	95.92%	96.24%	0.94
Fold 5	96.37%	95.54%	94.79%	95.16%	0.92
Fold 6	93.23%	97.38%	96.18%	96.78%	0.94
Fold 7	96.35%	96.45%	96.93%	96.69%	0.92
Fold 8	97.84%	95.91%	93.96%	94.92%	0.91
Fold 9	96.62%	95.65%	97.19%	96.41%	0.92
Fold 10	97.87%	95.53%	94.14%	94.83%	0.93
**Average**	**96.43%**	**96.08%**	**95.53%**	**95.80%**	**0.93**

**Table 4:** Classification results of scenario 3.

Fold	Accuracy	Precision	Recall	F1-Score	AUC
Fold 1	98.93%	97.30%	97.77%	97.53%	0.96
Fold 2	96.76%	94.46%	96.25%	95.35%	0.95
Fold 3	95.57%	95.83%	94.55%	95.19%	0.94
Fold 4	97.31%	96.97%	96.72%	96.84%	0.94
Fold 5	97.84%	94.22%	95.92%	95.06%	0.93
Fold 6	96.69%	96.02%	95.00%	95.51%	0.95
Fold 7	95.98%	94.77%	94.06%	94.41%	0.94
Fold 8	97.30%	95.55%	98.27%	96.89%	0.95
Fold 9	97.92%	97.09%	94.17%	95.61%	0.95
Fold 10	98.43%	98.18%	94.81%	96.47%	0.94
**Average**	**97.27%**	**96.04%**	**95.75%**	**95.89%**	**0.95**

**Table 5:** Results of the classification process performed before and after data augmentation.

Data Augmentation	Accuracy			AUC		
	Scenario 1	Scenario 2	Scenario 3	Scenario 1	Scenario 2	Scenario 3
Yes (3560 images)	95.77%	96.43%	97.27%	0.92	0.93	0.95
No (712 images)	87.56%	88.23%	88.65%	0.86	0.88	0.88

**Table 6:** Comparison of this study with the studies carried out in the literature.

Reference	Deep Learning Model	Accuracy
[4]	ResNet	98.80%
[7]	VGG16	88.89%
[8]	CNN	92.73%
**This study**	**ViT**	**95.77%**

## Data Availability

The datasets analyzed during the current study are available from the study: https://www.nature.com/articles/s41597-020-0360-7. Accessed on 11 December 2023.
